# Real-life effectiveness of sacubitril/valsartan in older Belgians with heart failure, reduced ejection fraction and most severe symptoms

**DOI:** 10.1038/s41598-024-64243-w

**Published:** 2024-06-12

**Authors:** Eléonore Maury, Ann Belmans, Kris Bogaerts, Stefaan Vancayzeele, Mieke Jansen

**Affiliations:** 1 Medical Department, Novartis Pharma NV/SA, Medialaan 40 Bus 1, 1800 Vilvoorde, Belgium; 2 Department of Public Health and Primary Care, I-BioStat, KU Leuven, Leuven, Belgium; 3 UHasselt, I-BioStat, Hasselt, Belgium

**Keywords:** Chronic heart failure, Registry, Real-world, Long-term observational study, Electronic healthcare datasets, Sacubitril/valsartan, Cardiology, Health care, Signs and symptoms

## Abstract

We assessed the real-world effectiveness of sacubitril/valsartan in patients with chronic heart failure (HF) and reduced ejection fraction (HFrEF) with an emphasis on those with older age (≥ 75 years) or with New York Heart Association (NYHA) class IV, for whom greater uncertainty existed regarding clinical outcomes. We conducted a retrospective cohort study based on patient-level linkage of electronic healthcare datasets. Data from all adults with HFrEF in Belgium receiving a prescription for sacubitril/valsartan between 01-November-2016 and 31-December-2018 were collected, with a follow-up of > 6 years. The total study population comprised 5446 patients, older than the PARADIGM-HF trial participants, and with higher NYHA class (all P < 0.0001). NYHA class improved following sacubitril/valsartan initiation (P < 0.0001 baseline vs. reassessment). Most concomitant medications were reduced. Remarkably, the risk of hospitalization for a cardiovascular reason and for HF was reduced by > 26% in the overall cohort, and in subgroups of patients ≥ 75 years, with NYHA class III/IV (all P < 0.0001) or with NYHA class IV (P < 0.05), vs. baseline. All-cause mortality did not increase in real-world patients with NYHA class III/IV. The results support the long-term beneficial effects of sacubitril/valsartan in older patients and in those experiencing the most severe symptoms.

## Introduction

The introduction of class of Angiotensin Receptor–Neprilysin Inhibitor (ARNI) has been a breakthrough in the pharmaceutical management of chronic Heart Failure (HF) with reduced ejection fraction (HFrEF). In 2014, the Prospective comparison of ARNI with Angiotensin-Converting-Enzyme Inhibitor (ACEI) to Determine Impact on Global Mortality and morbidity in Heart Failure (PARADIGM-HF) trial terminated early because of significant benefits of the ARNI sacubitril/valsartan, which contains the neprilysin inhibitor (NEPI) sacubitril and the Angiotensin Receptor Blockers (ARB) valsartan^1^. The treatment was namely more effective in reducing the physical limitations of HF, hospitalization for HF, and the risk of death from cardiovascular causes than ACEI enalapril^1^. Hence, the American College of Cardiology/American Heart Association (ACC/AHA) Task Force on clinical practice guidelines recommended the replacement of ACEI or ARB by ARNI for patients with chronic symptomatic New York Heart Association (NYHA) functional classification II/III HFrEF to further reduce morbidity or mortality^2,3^. Yet, the generalizability of randomized controlled trials in HFrEF remains questionable, because of the bias induced by criteria for enrollment and differences in clinical outcomes^4,5^.

This study aimed at providing insights into real-world clinical management of patients with HFrEF treated with sacubitril/valsartan in Belgium. We addressed uncertainties about the effectiveness of sacubitril/valsartan by measuring hospitalization and mortality rates during a long-term follow-up period. Specifically, there was still limited clinical experience in patients ≥ 75 years of age and in patients with NYHA class IV^6–8^.

## Methods

### Study population

This study was a nationwide, non-interventional, retrospective cohort study based on patient level linkage of integrated electronic healthcare datasets including administrative payer claims, national health registries, pharmacy claims and medical records. Reimbursement conditions for treatment initiation were the following: (i) treatment of adult patients (≥ 18 years) with symptomatic HFrEF and (ii) NYHA class II, III or IV, (iii) Left ventricular Ejection Fraction (LVEF) ≤ 35% assessed by echocardiography, (iv) prior treatment with ACEIs or ARBs, (v) initiation and prolongation of sacubitril/valsartan by a cardiologist or internist^9^.

Data from all adult patients with chronic symptomatic HFrEF in Belgium with a prescription for sacubitril/valsartan between 01-November-2016 and 31-December-2018, regardless of prior history or length of follow-up, were collected by Sciensano (healthdata.be platform)^9^ or by the national federation of independent pharmacists Algemene Pharmaceutische Bond (APB), comprising a total of 5446 patients, i.e., ~ 3% of patients living with chronic symptomatic HF in Belgium^9^. Pseudonymized data were gathered into a registry of real-world data (“Registry”). Full information about the technical architecture of the platform^9^, description of the databases and variables can be found in the Supplementary Methods S1. Briefly, the number of requests for sacubitril/valsartan and other co-medications and the evolution of NYHA class between 01-November-2016 and 31-December-2018 were determined. Hospitalization and mortality rates were analyzed with censoring dates of 31-December-2019 and 16-March-2023, respectively.

This non-interventional study with secondary use of data was performed in accordance with relevant guidelines and regulations^9^. The collection of pseudonymized data and its use was approved by the Belgian Data Protection Authority. The need for informed consent was deemed unnecessary according to national regulations (Belgian Data Protection Authority). All the protocols including the collection of data were in accordance with the European general data protection regulations. The purpose of this study and data processing were detailed in a privacy notice made available to the healthcare professional community in Belgium, aimed at informing patients treated with sacubitril/valsartan, per request of the Belgian Data Protection Authority.

### Post-hoc analysis of PARADIGM-HF trial

Full details of the trial design, entry criteria, and main results have been previously reported^1,10,11^. De-identified patient-level data of the trial were made available through Novartis Data42 platform using Palantir-Foundry architecture. The analyses were performed on 4187 patients in the sacubitril/valsartan arm who underwent valid randomization, unless otherwise specified.

### Statistical analyses

All-Cause Mortality Rates: The annual death rate and corresponding 95% confidence interval (CI) was calculated by means of a Poisson regression, using each patient’s follow-up time as offset. In addition, a Kaplan–Meier (KM) curve was calculated, whereby pointwise 95% CIs were calculated using the log(-log)-transformation for the standard error.

Hospitalization Rates: Annual hospitalization rates with corresponding 95% CIs were determined based on the number of hospitalizations per patient, with a Poisson regression, using the period at risk of each patient as offset.

The other results were either presented descriptively (i.e., prevalence) or represented as mean ± SD for group comparison. Two-tailed unpaired Student’s *t* test or Mann–Whitney test were applied to compare two independent groups, as appropriate. Comparisons between different conditions within a same group were made using two-tailed paired Student’s *t* test. For categorical variables (NYHA class), comparisons were performed with the use of Fisher’s exact test. Statistical tests were assessed at a 2-sided significance level of 5%. No corrections were made to the significance level for multiple testing due to the exploratory nature of the study. Analyses were conducted using SAS Enterprise guide 7.1 or Prism 9.3.1 GraphPad Software.

## Results

### Characteristics of the patients

The total study population comprised 5446 patients, 4 years older than the PARADIGM-HF trial participants (Mean ± SD, 67.8 ± 12.1 *vs*. 63.8 ± 11.5 years, P < 0.0001), and with higher NYHA class at baseline (43.3% *vs*. 23.2% with NYHA class III, 5.6% *vs*. 0.8% with NYHA class IV, but 0.0% *vs*. 4.3% with NYHA class I and 51.1% *vs.* 71.7% with NYHA class II, respectively, all P < 0.0001, Table 1, with 60.1% of patients having a recorded NYHA status). In contrast with randomized patients, real-world patients ≥ 75 years were more likely categorized as NYHA class III (51.3% *vs*. 30.2%, Table 1) consistently with the older age of those with NYHA class III or IV in the registry (Table S1). There was a male preponderance in both cohorts (75.4% in the registry *vs.* 79.0% in the trial, Table 1).

**Table 1:** Baseline characteristics of real-world patients and PARADIGM-HF trial participants.

Demographics	Total population	≥ 75 years
**Belgian registry**		
Total N of patients	5408	1705
Age (years), mean ± SD	67.8 ± 12.1	80.7 ± 4.2
Age group (years) n/N (%)		
< 55	781/5405 (14.5%)	0/1705 (0.0%)
55–64	1160/5405 (21.5%)	0/1705 (0.0%)
65–74	1759/5405 (32.5%)	0/1705 (0.0%)
≥ 75	1705/5405 (31.5%)	1705/1705 (100.0%)
Sex, n/N (%)		
Male	4078/5408 (75.4%)	1218/1705 (71.7%)
Female	1330/5408 (24.6%)	487/1705 (28.6%)
Baseline NYHA, n/N (%)		
NYHAI	0/3283 (0.0%)	0/1112 (0.0%)
NYHAII	1677/3283 (51.1%)	453/1112 (40.7%)
NYHAIII	1423/3283 (43.3%)	571/1112 (51.3%)
NYHAIV	183/3283 (5.6%)	88/1112 (7.9%)
**PARADIGM-HF (sacubitril/valsartan arm)**		
Total N of patients	4187	784
Age (years), mean ± SD	63.8 ± 11.5	79.1 ± 3.5
Age group (years), n/N (%)		
< 55	838/4187 (20.0%)	0/784 (0.0%)
55–64	1273/4187 (30.4%)	0/784 (0.0%)
65–74	1292/4187 (30.9%)	0/784 (0.0%)
≥ 75	784/4187 (18.7%)	784/784 (100.0%)
Sex, n/N (%)		
Male	3308/4187 (79.0%)	588/784 (75.0%)
Female	879/4187 (21.0%)	196/784 (25.0%)
Baseline NYHA, n/N (%)		
NYHAI	180 /4180 (4.3%)	24/781 (3.1%)
NYHAII	2998/4180 (71.7%)	512/781 (65.6%)
NYHAIII	969/4180 (23.2%)	236/781 (30.2%)
NYHAIV	33/4180 (0.8%)	9/781 (1.2%)

Prior to treatment initiation, patients in both cohorts were treated with a broad range of medications, including ACEI, ARB, Beta-Blockers (BB) and Mineralocorticoid Receptors Antagonists (MRA), which were the 4 drug classes used as guideline-directed therapy for HFrEF to reduce mortality (Table S2)^2^. There was a higher utilization of antihyperglycemic and lipid-lowering medications, as well as therapies for obstructive airway disease, in the registry (Table S2).

We further determined the etiology of heart disease by examining the overall hospital diagnoses in real-world (Table S3, *left*). Among 5408 patients, 71.8% were hospitalized for heart disease, 46.6% hospitalized for HF. A total of 49.6% patients was diagnosed with an ischemic etiology. Non-ischemic heart disease included hypertensive cardiomyopathy (21.4% of patients), dilated cardiomyopathy, alcoholic, drug-induced cardiomyopathy, or unspecified cardiomyopathy (14.4%), and non-ischemic rheumatic or congenital heart disease (1.8%). Atrial fibrillation was found in 11.1% of patients and other arrhythmias in 13.2% of patients. Other cardiovascular diagnoses were made, including hypertension (22.0% of patients), peripheral artery disease (5.8%) and cerebrovascular disease (2.9%) (Table S3, *left*).

The high prevalence of ischemic etiology was in line with the results of PARADIGM-HF trial^1^. Of note, the benefit of sacubitril/valsartan is expected to be consistent across the different etiologic categories^12^.

Lastly, real-world patients also presented a broad range of comorbidities such as respiratory diseases, type 2 diabetes, or primary malignancies, as attested by the hospital diagnoses. Prior hospital diagnoses showed that comorbidities increasing the risk of development of cardiovascular disease were frequent (Table S3, *right*), consistently with the prior medications described in Table S2.

### Prescriptions for sacubitril/valsartan and evolution of concomitant medications

**Figure 1:**
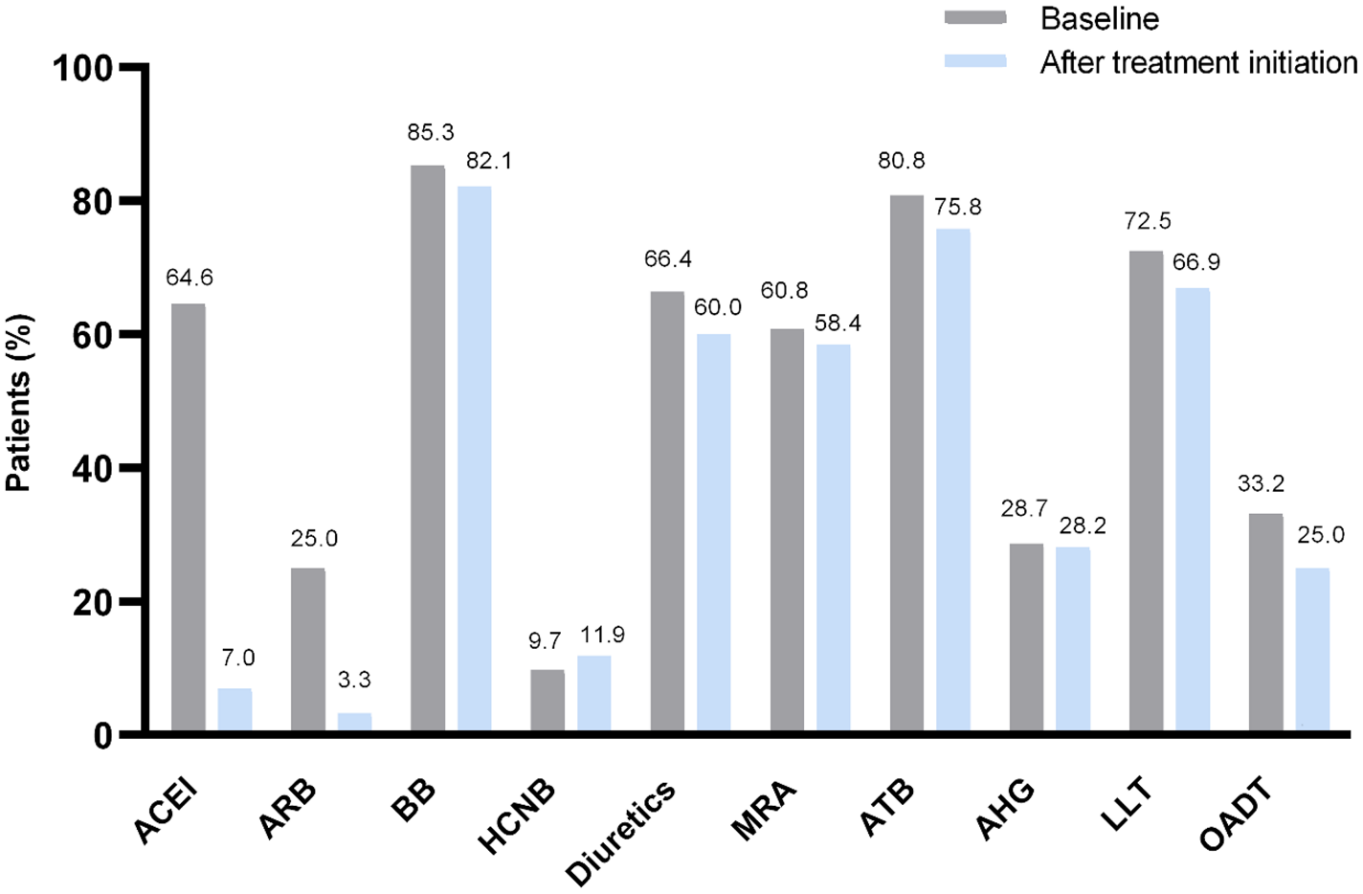
Decreased use of concomitant pharmaceutical therapies after initiation of sacubitril/valsartan in the Belgian registry. The percentage of patients receiving the following medications is displayed: *ACEI* angiotensin-converting-enzyme inhibitors, *ARB* angiotensin receptor blockers, *BB* beta-blockers, *HCNB* hyperpolarization-activated cyclic nucleotide gated cation (HCN) channel blockers, *Diuretics*, *MRA* mineralocorticoid receptors antagonists, *ATB* antithrombotics, *AHG* antihyperglycemics, *LLT* lipid-lowering therapy, *OAD therapy (OADT)* pharmacotherapy of obstructive airway disease.

Following patients’ characterization, we monitored the number of reimbursement requests for sacubitril/valsartan and concomitant pharmaceutical therapies (Table S4, Fig. 1). The percentage of patients obtaining a single authorization for reimbursement between 01-November-2016 and 31-December-2018 was higher for patients 75 years or older (70.8%) and for those with NYHA class IV (73.2%) (Table S4). During this timeframe, 39% of patients were prescribed the lowest dose of sacubitril/valsartan (24/26 mg) only, 37% the median dose (49/51 mg) and 24% the highest dose (97/103 mg). Sacubitril/valsartan utilization was associated with a reduced use of most concomitant therapies during this period, including other HF medications (Fig. 1). Consistent with the practice of administering sacubitril/valsartan in place of ACEI or ARB (but in conjunction with other HF therapies)^2^, the use of ACEI and ARB was decreased by ~ 9 and eightfold, respectively (Fig. 1). The continued utilization of ACEI and ARB may be attributed to their off-label use in combination with sacubitril/valsartan or it could be explained by administrative delays. Overall, our results suggest that the treatment led to an overall favorable cardio-renal profile in this population.

### Evolution of NYHA functional classification during treatment

**Figure 2:**
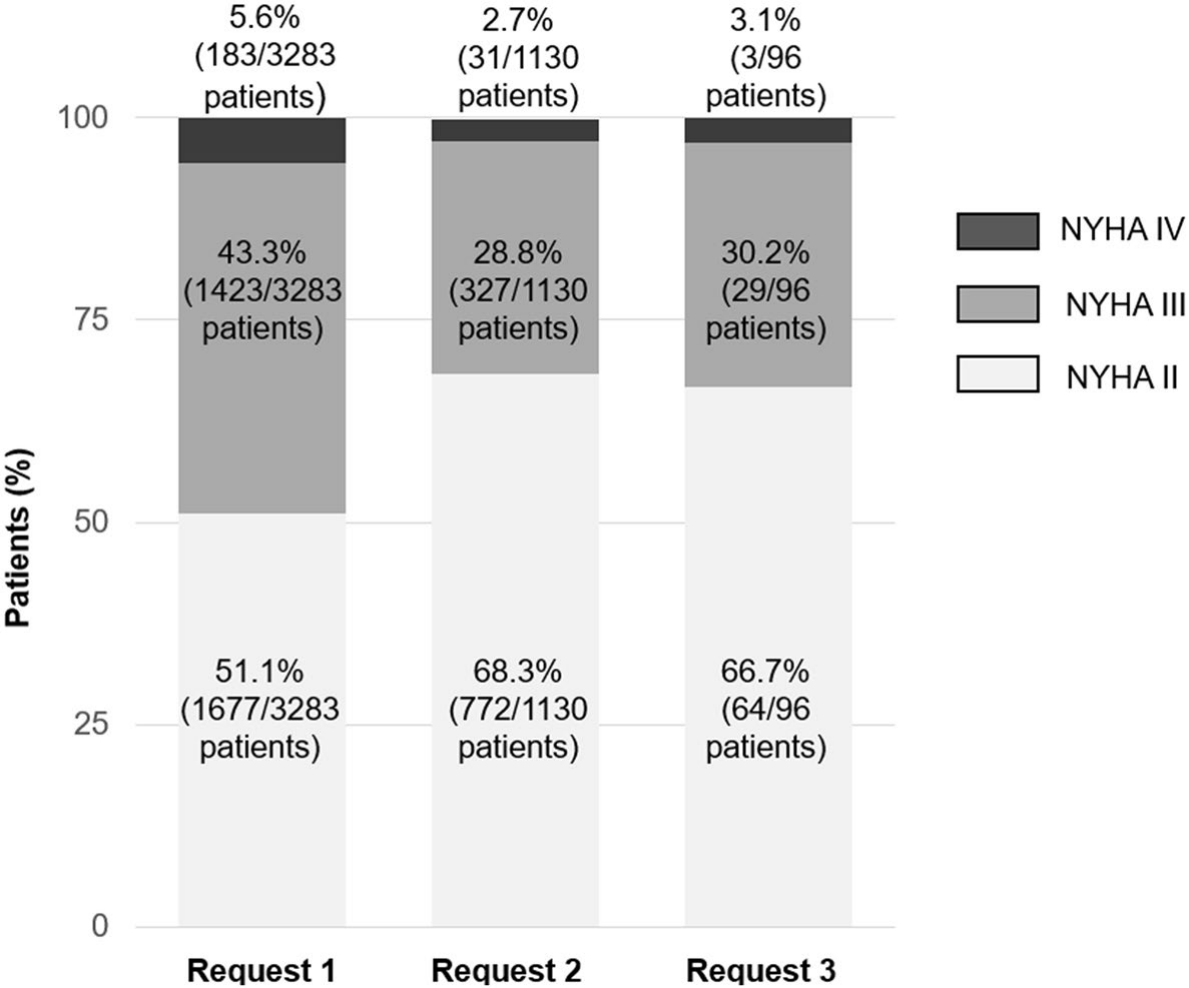
Improvement in New York Heart Association class with sacubitril/valsartan in a Belgian real-life clinical setting. NYHA status is first assessed before treatment initiation (baseline, first request for reimbursement) and is reassessed at subsequent requests. The absolute number of patients and percentages are provided.

Then, we investigated whether NYHA class improved with treatment. Remarkably, we found a ~ 1.5- and 2-fold decrease in the percentage of patients with NYHA class III and IV, respectively, between the first and the second reimbursement request (*i.e*., baseline *vs.* 12 months of treatment), associated with an increase in patients with NYHA class II (by 34%) (P < 0.0001, Fig. 2), a condition remaining stable when NYHA class was reassessed at the third request (12 *vs.* 24 months, not significant, Fig. 2). Collectively, between the first and the third reimbursement request, 21.9% of patients displayed a clinical improvement in NYHA class (Table S5). NYHA class was unchanged in 76.0% of patients and worsened in only 2.1% (Table S5). The identical annual mortality rate in patients with NYHA class III/IV at baseline and in the overall cohort (see Sect. Mortality data) further suggests that the treatment directly improves HF symptoms.

### Evolution of the number of hospitalizations during treatment

Then, we examined whether we could confirm the reduced risk of hospitalization for a CV reason or for HF, key study outcomes in PARADIGM-HF. In the overall cohort, during treatment (until 31-December-2019), the annual hospitalization rate for any reason was 1.70 hospitalizations per year (95% CI 1.64–1.77), and 0.55 hospitalizations per year for cardiovascular causes (95% CI 0.53–0.58) (Table 2). The annual HF hospitalization rate was 0.25 hospitalizations per year (95% CI 0.23–0.27) (Table 2).

**Table 2:** Number of hospitalizations for any reason, CV or HF-related hospital admissions and years at risk after start of sacubitril/valsartan in the Belgian registry.

	Hospitalizations for any reason	CV hospitalizations	HF hospitalizations
**Number of hospitalizations per patient**			
[n] Mean (SD)	[5381] 2.9 (4.0)	[5383] 1.0 (1.5)	[5389] 0.4 (1.0)
Median (Q1; Q3)	2.0 (1.0; 4.0)	0.0 (0.0; 1.0)	0.0 (0.0; 1.0)
Min–Max	0–53	0–17	0–14
**Years at risk per patient**			
[n] Mean (SD)	[5381] 1.73 (0.71)	[5383] 1.75 (0.71)	[5389] 1.76 (0.71)
Median (Q1; Q3)	1.72 (1.22; 2.27)	1.73 (1.23; 2.28)	1.74 (1.24; 2.29)
Min–Max	0.01–3.15	0.00–3.15	0.00–3.15
**Total number of hospitalizations (N)**	15,861	5220	2383
**Total time at risk [years] (sum)**	9331.70	9431.74	9482.11
**Annual hospitalization rate (hospitalizations/year) [est. (95% CI)]**	1.70 (1.64; 1.77)	0.55 (0.53; 0.58)	0.25 (0.23; 0.27)

*HF* heart failure, *CV* cardiovascular.

To further confirm clinical benefit and value of sacubitril/valsartan, we determined the annual hospitalization rates for cardiovascular disease and for HF specifically, before treatment initiation, *i.e.*, based on the number of hospitalizations per patient from day 1 in the registry (01-November-2016) until treatment start, and compared it to the annual hospitalization rates during treatment (until 31-December-2019, Table 3). Strikingly, the treatment induced a very significant decrease in the annual hospitalization rate by more than 26% for both CV and HF reasons in the overall cohort (Table 3, P < 0.0001 vs. before sacubitril/valsartan).

**Table 3:** Decrease in cardiovascular or heart-failure hospitalizations in patients with HFrEF treated with sacubitril/valsartan in real‐world clinical setting in Belgium.

	Total population (N = 5408)	Baseline ≥ 75 years (N = 1705)	Baseline NYHA III/IV (N = 793)	Baseline NYHA IV (N = 75)
**CV hospitalizations before sacubitril/valsartan**				
Annual CV hospitalization rate (hospitalizations/year), Est. (95% CI)	0.75 (0.72; 0.77)	0.72 (0.68; 0.76)	0.72 (0.66; 0.78)	0.79 (0.62; 1.01)
**CV hospitalizations after sacubitril/valsartan**				
Annual CV Hospitalization Rate (Hospitalizations/year), Est. (95% CI)	0.55 (0.53; 0.58)	0.52 (0.47; 0.58)	0.50 (0.45; 0.56)	0.49 (0.36; 0.67)
P value	< 0.0001	< 0.0001	< 0.0001	< 0.05
**HF hospitalizations before sacubitril/valsartan**				
Annual HF hospitalization rate (hospitalizations/year), Est. (95% CI)	0.34 (0.32; 0.36)	0.36 (0.33; 0.39)	0.34 (0.30; 0.38)	0.36 (0.26; 0.49)
**HF hospitalizations after sacubitril/valsartan**				
Annual HF hospitalization rate (hospitalizations/year), Est. (95% CI)	0.25 (0.23; 0.27)	0.28 (0.24; 0.33)	0.20 (0.17; 0.23)	0.21 (0.13; 0.32)
P value	< 0.0001	< 0.0001	< 0.0001	< 0.05

*HF* heart failure, *CV* cardiovascular.

There is greater uncertainty regarding clinical outcomes in patients ≥ 75 years of age and with NYHA class IV at baseline due to the limited representation of these patients in the pivotal trial, and real-world evidence evaluating the clinical outcomes of sacubitril/valsartan treatment in these groups is still scarce^6–8^. Here, we observed that treatment with sacubitril/valsartan consistently led to a significant decrease in the annual hospitalization rate for both CV and HF across all subgroups including patients ≥ 75 years or with NYHA class III/IV at baseline (P < 0.0001 vs. before treatment, Table 3). In the smaller subgroup of patients with NYHA class IV at baseline, the decrease was the highest, reaching ~ − 38% for CV hospitalizations and ~ − 43% for HF-hospitalizations (P < 0.05 vs. before treatment, Table 3).

Thus, our results show a beneficial effect of sacubitril/valsartan on CV and HF hospitalizations across all subgroups, including older individuals and patients displaying the most severe clinical symptoms (Table 3).

### Mortality data

A total of 2056 patients out of 5408 died in the period between 01-November-2016 and 16-March-2023. The mean age (± SD) of the deceased patients was 75.9 years (± 10.0), with 1036 patients < 75 years of age and 1020 patients ≥ 75 years. Deceased patients were older in the Belgian registry than in the sacubitril/valsartan arm of PARADIGM-HF (67.0 ± 12.4 years in the trial, P < 0.0001).

In the Belgian registry, mortality due to any reason did not increase with the severity of NYHA, as 37.8% of patients in NYHA class III/IV at baseline died, against 38.0% for the whole cohort (Table 4). The mean ± SD length of follow-up was 4.11 ± 1.71 years (49 months), with a median of 4.65 years (56 months) for this cohort. The overall annual death rate was 0.09 deaths/year (95% CI 0.08–0.10), which was almost doubled for patients ≥ 75 years of age, but unchanged in patients with NYHAIII/IV at baseline (0.09 deaths/year, 95% CI 0.07–0.10, Table 4).

**Table 4:** Length of follow-up per patient treated with sacubitril/valsartan and annual death rate in real‐world clinical setting in Belgium.

All-cause mortality	Total population	Baseline ≥ 75 years	Baseline NYHA III/IV
Number of deaths, n/N (%)	2056/ 5405 (38.04%)	1020/1705 (59.89%)	300/793 (37.83%)
Age at death (years), [n] mean (SD)	[2056] 75.9 (10.0)	[1020] 83.8 (4.5)	[300] 77.6 (9.6)
Number of requests at time of death, n/N (%)			
1	761/1067 (74.13%)	369/493 (74.85%)	229/300 (76.33%)
2	270/1067 (25.30%)	122/493 (24.75%)	71/300 (23.67%)
3	6/1067 (0.56%)	2/493 (0.41%)	0/300 (0.00%)
Length of follow-up per patient (years), [N] mean (SD)	[5405] 4.11 (1.71)	[1703] 3.39 (1.88)	[793] 4.41 (1.25)
Median (Q1; Q3)	4.65 (3.24; 5.31)	4.11 (1.61; 4.93)	4.63 (3.88; 5.28)
Min–Max	0.00–6.43	0.00–6.35	0.15–6.35
Total follow-up time (years), N	22,233.75	5781.09	3496.50
Annual death rate (deaths/year), Est. (95% CI)	0.09 (0.08; 0.10)	0.18 (0.15; 0.21)	0.09 (0.07; 0.10)

The duration of follow-up in the Belgian registry was longer than that of the PARADIGM-HF trial, for which the median duration was 2.25 years (27 months) and patients in both arms were treated for up to 4.3 years^1^, thereby enabling comparison of the estimates of the survival function. Of note, follow-ups on mortality in the present registry partially occurred during the SARS-CoV-2 pandemic.

A total of 1422 patients from the Belgian registry had died at 1260 days, which may correspond to June 2022 for patients with a first prescription in December 2018. This results in a KM-estimate of cumulative mortality of 26% in the registry (*vs.* 1277 patients in PARADIGM-HF, with KM-estimate of cumulative mortality of 24%), resulting in a relative difference of < 9% between both cohorts, as presented in Fig. S1^1^. A similar relative difference was found at 1195 days after treatment start [~ 9% (relative), with 25.4% in the registry vs. 23.3% in the trial].

The estimate of the survival function during a follow-up period of > 6 years is provided in Fig. S2.

## Discussion

This study demonstrated the long-term treatment benefits of sacubitril/valsartan in real-world clinical practice, in a heterogeneous cohort of patients with HFrEF, with consistent results across subgroups including older and frailer patients. Specifically, a significant reduction in the risk of both hospitalizations for a cardiovascular reason and for HF by > 26% was observed after initiation of sacubitril/valsartan treatment, in the overall cohort but also specifically in patients ≥ 75 years of age, and in patients with NYHA class III/IV or with NYHA class IV. This high reduction in real-world setting (by comparison with ~ 21% *vs*. enalapril in the pivotal trial)^1^, is in line with previous reports^5,13,14^. In addition, real-world patients exhibited an improvement in NYHA class and a decreased use of most medications for HF and other relevant concomitant pharmaceutical therapies following treatment initiation.

The difference in demographics, comorbidities and co-medications between the patients enrolled in the registry and those recruited in the pivotal trial might be explained by the restrictive CV exclusion criteria in the trial (*e.g*., acute coronary syndrome, stroke, transient ischemic attack, major cardiovascular surgery). Yet, ischemic heart disease, a common cause of HF^3^, was a major cause for HF in both cohorts.

We observed a slight increase in all-cause mortality in the real-world registry as compared with that seen in the pivotal trial [< 9% (relative) at 1200–1260 days after treatment initiation], which might be partially due to the impact of SARS-CoV-2 pandemic. Patients with HF who contract SARS-CoV-2 infection are at a higher risk of cardiovascular and non-cardiovascular morbidity and mortality^15^. The difference might also be explained by the older age at baseline, in line with previous analyses of the trial showing that the magnitude of long-term benefits and the duration of survival is smaller in older patients^16^. Age is known to be an independent predictor of short (30 days) and long (5–8 years) term mortality in patients with HFrEF^17^. Advancing age significantly impacts mortality, with a gain of 10 years increasing the risk by 23% and 55% for short- and long-term mortality, respectively^17,18^. Moreover, the NYHA functional classification at baseline, which was also higher in this registry, is another well-established independent predictor of mortality^18,19^. It has been described that patients with NYHA class II, III and IV have an increased mortality risk of 54%, 156% and 846%, respectively, compared with NYHA class I patients^20^.

Furthermore, the prevalence of comorbidities known to increase the risk of mortality in patients with HFrEF, such as type 2 diabetes^21,22^ or chronic obstructive pulmonary disease^23,24^, was higher in the registry, as attested by the elevated use of respective therapies. Lipid-lowering therapies were also highly prescribed. The prescription rate of pharmacological agents was at a comparable level or higher to that of most real-world evidence studies, except for diuretics^4^.

Despite the older age and frailty of patients with HFrEF and the concurrent SARS-CoV-2 pandemic, the risk of death from any cause in real-world was only slightly higher than that seen in the pivotal trial.

Taken together, our study confirms that older and highly medicated patients can benefit from sacubitril/valsartan in clinical practice. This work complements other recent real-world studies filling the gap of information regarding safety of sacubitril/valsartan in patients above 75 years^25,26^.

Limitations: Our study includes several limitations. The databases used in this analysis were not designed for research purposes but for reimbursement (of medicine or hospital activities) or for administrative purposes. Missing data is a common bias in studies based on secondary use of data^5^. Bias may also arise from administrative delays, namely relating to reimbursement requests or hospitalizations data, or not having registered eligible patients who died before taking sacubitril/valsartan (survival bias). Patient-level data were restricted to individuals with a prescription for sacubitril/valsartan. The study lacked a control group, comprising patients who did not receive sacubitril/valsartan on top of standard of care, important for establishing a reliable baseline to evaluate the true effectiveness of the intervention. Moreover, the analysis of numbers of requests for reimbursement and of the evolution of NYHA class was restricted to a short period (up to 2.2 years), and some clinical characteristics such as LVEF were not available. The titration regimen and the exact percentage of patients discontinuing the study medication (e.g., tolerability issues, patient preferences, pregnancy) could not be determined in this study. Given the nature of our study as an observational and non-interventional investigation, the patients had full autonomy to navigate their adherence to medication. Lastly, guideline-directed medical therapy has evolved since the initiation of this study and now includes sodium-glucose cotransporter-2 inhibitors^3^.

Despite limitations*,* these results strongly suggest a consistent response to sacubitril/valsartan in real-world clinical practice for older and frailer patients with chronic HFrEF.

### Supplementary Information

Supplementary Information.

## Data Availability

Additional information is available from the corresponding author (eleonore.maury@novartis.com) upon reasonable request. The information presented in this paper combines several Belgian administrative databases. The data use is subject to the European Union's General Data Protection Regulation. Patient-level data were available via the healthdata.be in the framework of a managed entry agreement between Novartis and the payer institute National Institute for Health and Disability Insurance (NIHDI-RIZIV-INAMI). Access to data was restricted to address uncertainties in the context of a managed entry agreement and related publication. Patient-level data may not be shared publicly or transferred due to ethical reasons and security considerations.
