# Supplementary material for: Real-life effectiveness of sacubitril/valsartan in older Belgians with heart failure, reduced ejection fraction and most severe symptoms
Source: Sci Rep. 2024 Jun 12;14:13512. doi: 10.1038/s41598-024-64243-w (PMC11169478; doi:10.1038/s41598-024-64243-w)
Supplement: Supplementary file 1 — Supplementary Information. [file 41598_2024_64243_MOESM1_ESM.pdf]

Real-life effectiveness of sacubitril/valsartan in older Belgians with heart failure, reduced ejection fraction and most severe symptoms

Authors: Eléonore Maury, Ann Belmans, Kris Bogaerts, Stefaan Vancayzeele†, Mieke Jansen

Supplementary Material

Supplementary Methods:

1. Data sources and variables

a. Healthdata.be data network platform

The data for this study, restricted to the reimbursement of sacubitril/valsartan (commercially named Entresto®), was retrieved by the Belgian Institute Sciensano. Each reimbursement request covers a period of 364 days [1].

Sciensano and the payer institute National Institute for Health and Disability Insurance (NIHDI-RIZIV-INAMI) have joint responsibility for the electronic data capture system named healthdata.be with the aim to facilitate data exchange between healthcare professionals and researchers to increase public health knowledge and to adjust health care policy. Healthdata.be is a data network platform that coordinates and interconnects the electronic exchange of healthcare data in all regions of Belgium comprising integrated information from different healthcare stakeholders. The following databases were used:

- National Intermutualistic College (NIC) database: The NIC is an organization in which all Belgian Sickness Funds are represented including the representatives of the five national federations of the sickness funds, the Health and Invalidity Aid Fund and the National Accident Care Center for Medical Care. Databases "Agreements" cover the agreements made between health care providers and sickness funds (i.e., health insurance companies) and are collected from the Belgian sickness funds and include all authorizations from the advisory physician for each medicine in the chapter IV. The application for reimbursement of these Chapter IV medicines is managed by the "Chapter IV Agreement Requesting System" or "CIVARS" application of the NIC. Agreements include the following: Pseudo-Identification of the Beneficiary, Prescription of sacubitril/valsartan, First Request for Refund and Renewal, patient condition:

NYHA Classification II, III or IV. It is important to mention the following limitations: i) providing the NYHA status was not mandatory for subsequent reimbursement requests and was therefore underreported, ii). reimbursement requests have only been reported until December 31, 2018.

- The National Register of Natural Persons (NRNP) database originates from the Federal Public Service. The data have been collected between November 1, 2016 and March 16, 2023 and include NISS, Gender, birth month and year, Address (city), Date and Place of Death.

- Intermutualistic Agency (IMA) database: IMA is a platform where data from the health funds is brought together and analyzed. The collection of data is done through the public pharmacies, pricing services of the pharmacists and insurance institutions. However, no data on patient identification, medical records and diagnosis are available. Data from IMA includes sacubitril/valsartan prescribed and dispensed to the individual patient with HFrEF, Prescription Date, Dose and Package Size.

- Minimal Hospital Data (MHD) database: For financial reimbursement purposes, Belgian hospitals need to comply with compulsory registration of the Databases MDH. These data include the International Classification of Diseases, Clinical Modification, version 10 (ICD-10-CM) coding of admission diagnoses and the ICD-10-PC for procedures. MDH are managed by the Federal Public Service (FPS) for Health, Food Chain Safety and Environment. MDH include the following: Verified Admission Diagnosis, Type of Discharge, Indicator Age, Code Re-admission, Gender, Code of Cause of Death, Code of Verified Admission Diagnosis, Zip code, Type of Hospitalization, Month/Year of Hospitalization, Month/Year of Discharge, Number of Fully/Non-/Partially Invoiced Days in the Previous and Current Registration Year, Year of Birth, Cause of Death, Code Diagnosis, Code Procedure and Date, Type of Test, Results and Date, Reason for Emergency Admission and Treatment, Invoice Details, Code Main and Secondary Diagnosis. Some hospitals might experience a delay in the registration of MHD.

- The IFSTAT Database is collected from the Institute for Pharmaco-Epidemiology in Belgium (IPhEB) via pharmacies (i.e., pricing services). This database contains data used by the invoicing offices to obtain reimbursement of the pharmaceutical supplies for the affiliated pharmacies and aims at showing that the beneficiaries of the compulsory health insurance in Belgium were delivered. Data are aggregated by district without enabling the potential identification of the prescriber or the patients. Data include Reimbursement

Category of Medicine, CNK Code Medicine, Delivery Date, Year and Month of Invoicing. Reimbursement can be requested up to 2 years after the procedure.

All the electronic data sources available at the time of data extraction were collected.

b. Databases from the national federation of independent pharmacists (APB)

APB data were also made available, independently from healthdata.be. A decentralized database managed by the pricing services (TariferingsDiensten, TD) and APB has been developed, to perform longitudinal studies, a project named BelPharData (80% of all Belgian pharmacies divided into 14 geographically clustered TDs). APB also provides data related to the delivery of medication, as for IPhEB. The TDs collect the data from its members and then sends it to the various insurance institutions to which the patients are affiliated. The insurance institutions in turn send the data to the NIHDI-RIZIV-INAMI. Part of the information from TD is also sent to the BelpharData database.

This database includes co-medication of 5446 patients who were delivered sacubitril/valsartan between November 1, 2016 and December 31, 2018. Anonymous aggregated data were provided without possible re-identification of patients, pharmacies or prescribers. Prior medications were determined with the same duration as the follow-up period after the initiation of sacubitril/valsartan.

Antihyperglycemic therapy includes metformin, sulfonylureas, thiazolidinediones, glinides, dipeptidyl peptidase 4, alpha-glucosidase, and insulin. Lipid Lowering Therapy (LLT) includes HMG CoA reductase inhibitors (mainly statins), Peroxisome proliferator-activated receptor α agonists, cholesterol absorption inhibitor, bile acid sequestrants, citrate lyase inhibitors, proprotein convertase subtilisin/kexin type 9 (PCK9) inhibitors. Hyperpolarization-activated cyclic nucleotide gated cation (HCN) channel Blockers (HCNB) include mainly ivabradine and ivabradine hydrochloride. Diuretics include thiazide-like chlortalidone, indapamide, and loop diuretics furosemide, bumetanide, torasemide. Calcium channels Blockers were not included in this analysis. Mineralocorticoid Receptors Antagonists (MRA) include mainly spironolactone and eplerenone, targeting aldosterone action. Antithrombotic drugs include platelet aggregation inhibitor, anticoagulants, thrombolytic drugs. Pharmaceutical management of obstructive airway disease include β2 adrenergic receptor agonist, muscarinic anticholinergic, glucocorticoids, leukotriene modifiers, theophylline,

anti-immunoglobulin E (IgE), anti-interleukins (IL) 4/13, anti-IL5/IL5 receptor monoclonal antibody, antitussives, mucolytics, expectorants. Anti-neoplastic agents, endocrine therapy, anti-dementia treatments and other less represented drug classes were not included in this analysis.

2. Analyses of the healthdata.be datasets

a. Overall number of patients, determination of age, date of treatment initiation and censoring date in the Belgian Registry

- Healthdata.be granted access to a first set of data in 2021, enabling to perform a first analysis. This first dataset (healthdata.be dataset 1) was used to determine the number of requests for sacubitril/valsartan and the evolution of NYHA functional classification during treatment. In 2023, healthdata.be provided access to a second set of data (healthdata.be dataset 2), corresponding to the extended data of the patients already present in the sacubitril/valsartan Registry. The analysis of healthdata.be dataset 2 was performed to determine the characteristics of patients with chronic HFrEF in real-world, as well as hospitalization rates and mortality rates.

Healthdata.be dataset 1 included a total of 5606 patients. From these 5606 patients the following were excluded i) the patients without records in the IMA database (*i.e.,* for whom there is no evidence that sacubitril/valsartan was dispensed), ii) patients without data besides IMA records (*i.e.,* no information regarding any follow-up), iii) patients who started the study medication on December 31, 2018 (*i.e*, cannot contribute any information), iv) patients whose Death Date in the NRNP is prior to sacubitril/valsartan initiation. The total number of patients included in the analyses was therefore N = 5317. An identical method was applied for the analysis of the healthdata.be dataset 2 data, therefore including N = 5408 out of the 5779 total patients.

For both analyses of healthdata.be dataset 1 and 2, the date of birth was based on NRNP for most patients, whereby the day of birth was assumed to be on the 15th of the recorded birth month and year. When not available (N=132 patients in the 1st analysis and N=117 in the 2nd analysis), the birth year of patient was retrieved from the hospitalization records. In these cases, the date of birth was imputed as July 01 of that

year. The first day of treatment or Day 0 was defined as the date of the first medication delivery. The age at start of treatment was calculated using the imputed dates of birth and start dates.

For analyses of healthdata.be dataset 1, the censoring date was December 31, 2018. For healthdata.be dataset 2, the censoring date for the analysis of hospitalization rates was December 31, 2019, and the censoring date for the analysis of mortality rates was March 16, 2023. The total follow-up time for the analysis of mortality rates was calculated as the number of days between Day 0 and the date of death (as retrieved from the NRNP database). Otherwise, the total follow-up time was the number of days between Day 0 and March 16, 2023.

b. Determination of Hospitalizations, Cardiovascular (CV) and HF Hospitalizations

Overlapping Hospital Records (*i.e.*, multiple discharge date with the same hospital admission date) were collapsed. In addition, consecutive hospital records with start date one day after the previous stop date were also collapsed unless the record was an admission to the emergency ward. After collapsing hospital records as describe above, a hospitalization was deemed to be due to CV or HF if the Diagnosis at Admission (ICD-10 code) of at least one of the collapsed records were related to CV or HF, respectively. Hospitalization records with special codes (*e.g.*, UUUUUU for emergency ward) or missing codes were deemed to be non-CV, non-HF. Hospital diagnoses were available for >85% of patients starting from 30-720 days prior to treatment and during treatment for all patients, the last day for hospitalization records being December 31, 2019.

The period at risk was calculated as the total follow-up time (from day 0, first day of treatment, to either December 31, 2019 or death, whichever comes first) from which the total number of in-hospital days was subtracted. Patients who first received sacubitril/valsartan in the hospital and died during the same hospitalization were not considered at risk for hospitalization and therefore not included in the analysis.

Supplementary Results:

Table S1. Baseline characteristics of patients with NYHA class III and IV in the Belgian registry and the PARADIGM-HF cohort

**Belgian registry**		
Demographics	*NYHA III*	*NYHA IV*
Total N of Patients	1423	183
Age (years) (Mean±SD)	70±11	72±13
Age group (years) (n, %)		
<55	150 (10.5%)	15 (8.2%)
55-64	246 (17.3%)	29 (15.8%)
65-74	456 (32.0%)	51 (27.9%)
≥75	571 (40.1%)	88 (48.1%)
Sex (n, %)		
Male	1077 (75.7%)	129 (70.5%)
Female	346 (24.3%)	54 (29.5%)

**PARADIGM-HF (Sacubitril/valsartan arm)**		
Demographics	*NYHA III*	*NYHA IV*
Total N of Patients	969	33
Age (years) (Mean±SD)	66±11	65±10
Age group (years) (n, %)		
<55	153 (15.8%)	3 (9.1%)
55-64	266 (27.5%)	10 (30.3%)
65-74	314 (32.4%)	11 (33.3%)
≥75	236 (24.4%)	9 (27.3%)
Sex (n, %)		
Male	724 (74.7%)	19 (57.6%)
Female	245 (25.3%)	14 (42.4%)
	969	33

Characteristics of patients in the sacubitril/valsartan arm of PARADIGM-HF are determined at randomization. Abbreviation: New York Heart Association (NYHA).

Table S2. Baseline medications of patients in the Belgian registry and in the PARADIGM-HF trial (% of patients in the respective cohort)

Medications	*Belgian registry (%)*	*PARADIGM-HF‡ (%)*
Angiotensin-Converting-Enzyme inhibitors (ACEI)	64.6	78.0
Angiotensin Receptor Blockers (ARBs)	25.0	22.2
Beta-Blockers (BB)	85.3	93.1
Diuretics	66.4	80.3
Mineralocorticoid Receptors Antagonists (MRA)	60.8	54.2
Antithrombotic drugs	80.8	
Oral anticoagulant		31.0
Antiplatelet agent		57.0
Antihyperglycemic therapy	28.7	25.5
Pharmaceutical management of dyslipidemia	72.5	56.6
Pharmaceutical management of obstructive airway disease	33.2	6.0

Abbreviations: Angiotensin-Converting-Enzyme inhibitors (ACEI), Angiotensin Receptor Blockers (ARBs), Beta-Blockers (BB), Mineralocorticoid Receptors Antagonists (MRA).

‡As determined from [2-5]

Table S3. Hospital diagnoses of patients with HFrEF in the Belgian registry

Hospital Diagnoses, N of Patients per Diagnosis	N of patients, Total diagnoses	N of patients, Diagnoses prior to treatment
Total N of Patients	5408	5408
Heart Disease, n (%)		
Heart Disease	3885 (71.84%)	2843 (52.57%)
Heart Failure	2519 (46.58%)	1701 (31.45%)
Ischemic Heart Disease	2682 (49.59%)	1875 (34.67%)
Non-Ischemic dilated cardiomyopathy, alcoholic or drug-induced cardiomyopathy, or unspecified	777 (14.37%)	377 (6.97%)
Non-Ischemic Hypertensive Cardiomyopathy	1158 (21.41%)	667 (12.33%)
Non-Ischemic Rheumatic or Congenital Heart Disease	97 (1.79%)	60 (1.11%)
Atrial Fibrillation and Flutter	599 (11.08%)	314 (5.81%)
Other Arrythmias (Etiology Unspecified)	713 (13.18%)	308 (5.70%)
Other Cardiovascular Disease, n (%)		
Peripheral Artery Disease	314 (5.81%)	135 (2.50%)
Cerebrovascular Disease	157 (2.90%)	85 (1.57%)
Pulmonary Vascular Disease or Oedema	104 (1.92%)	48 (0.89%)
Hypertension	1187 (21.95%)	682 (12.61%)
Other relevant comorbidities, n (%)		
Type 2 Diabetes	146 (2.70%)	56 (1.04%)
Renal Disease	852 (15.75%)	380 (7.03%)
Hyperkalemia	18 (0.33%)	4 (0.07%)
Dyslipidemia	1 (0.02%)	0 (0.00%)
Liver Disease	29 (0.54%)	11 (0.20%)
Obesity	39 (0.72%)	8 (0.15%)
Others comorbidities, n (%)		
COPD	236 (4.36%)	110 (2.03%)
Asthma	16 (0.30%)	9 (0.17%)
Hypotension	93 (1.72%)	23 (0.43%)
Primary Malignacy	586 (10.84%)	254 (4.70%)
Sleep Apnoea	320 (5.92%)	134 (2.48%)

Total hospital diagnoses (*left*) include all diagnoses between November 01, 2016 and December 31, 2019, while prior diagnoses (*right*) only include those occurring before treatment initiation (between 01-November-2016 and treatment start). Abbreviations: Heart Failure and reduced ejection fraction (HFrEF), chronic obstructive pulmonary disease (COPD).

Table S4. Number of reimbursement requests in the Belgian registry

Requests		*Total*	*≥75 Years*	*NYHA III*	*NYHA IV*	*NYHA III/IV*
Total N of patients		5180	1616	1423	183	1606
Request 1	n (%)	3425 (66.1%)	1144 (70.8%)	934 (65.6%)	134 (73.2%)	1068 (66.5%)
Request 2	n (%)	1619 (31.3%)	438 (27.1%)	443 (31.1%)	45 (24.6%)	488 (30.4%)
Request 3	n (%)	135 (2.6%)	33 (2.0%)	45 (3.2%)	4 (2.2%)	49 (3.1%)
Request 4	n (%)	1 (0.0%)	1 (0.1%)	1 (0.1%)	0 (0.0%)	1 (0.1%)

The absolute number of patients and percentage are provided. Abbreviation: New York Heart Association (NYHA).

In parallel to this information, between November 2016-December 2018, a total of 65.6% of patients exhibited high compliance, indicating that they had no treatment gap based on the supply of their last prescription, while 17.8% of patients stopped treatment (i.e., theoretical treatment gap of 3 months or more) as determined by the collected prescription information (by APB). The information in the pharmacy databases should be interpreted as a proxy for delivery, not for administration by a clinician or use by the patient.

Table S5. Changes in NYHA classification in the Belgian registry after treatment initiation

	Change in NYHA functional classification	
	Between Request 1 and Request 2	Between Request 1 and Request 3
	n/N (%)	n/N (%)
**Deterioration**	40/1130 (3.54)	2/96 (2.08)
**Stable**	866/1130 (76.64)	73/96 (76.04)
**Improvement**	224/1130 (19.82)	21/96 (21.88)

Abbreviation: New York Heart Association (NYHA).

Figure S1. Kaplan-Meier curve for death from any cause in the Belgian registry and comparison with the PARADIGM-HF trial

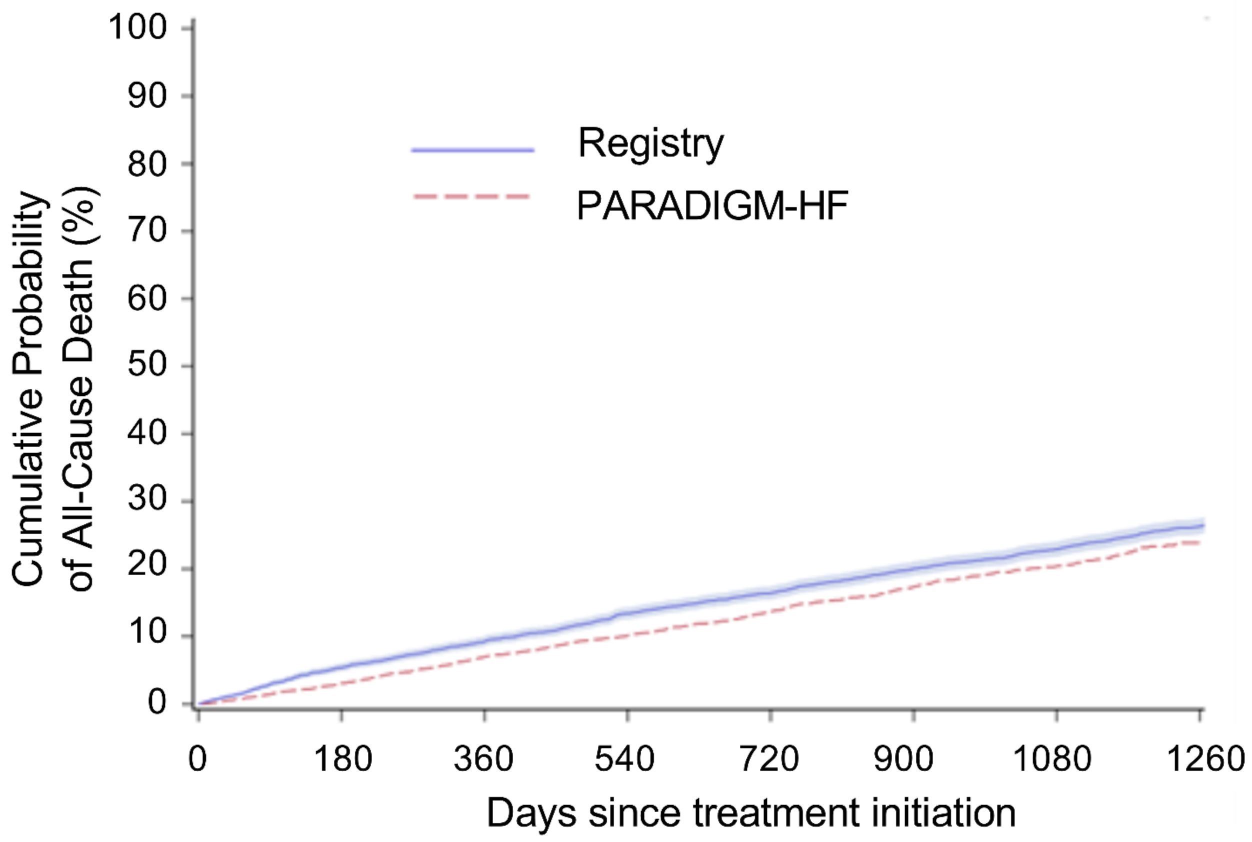

The figure illustrates the cumulative probability of death since the first prescription of study medication for real-world patients or since randomization for PARADIGM-HF patients. Lighter blue indicates the 95% confidence interval.

Figure S2. Kaplan-Meier curve for death from any cause in the Belgian registry, over a follow up period of > 6 years

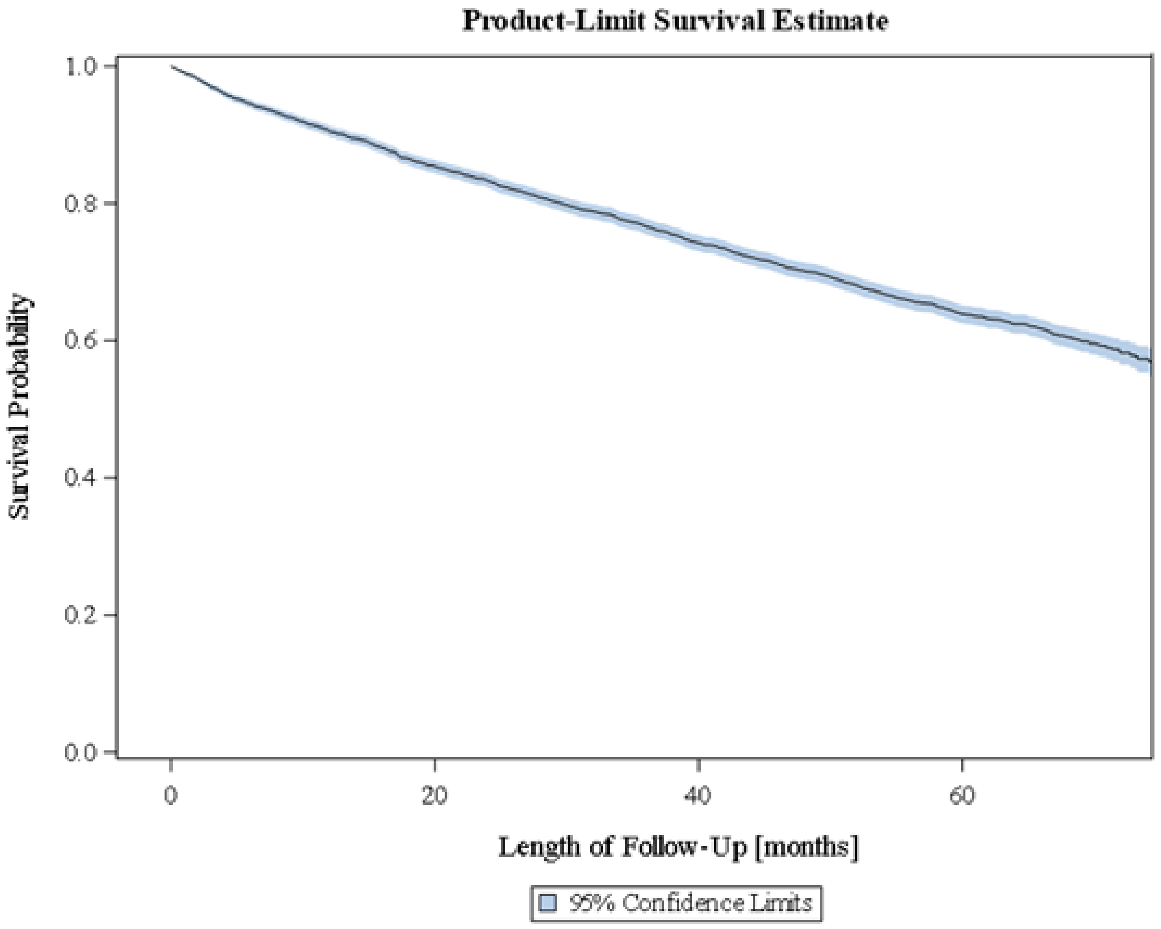

Lighter blue indicates the 95% confidence interval.

Supplementary References:

1. Infrastructure healthdata.be (accessed June 2019), https://www.ehealth.fgov.be/ehealthplatform/file/view/AWtQLJ1jgwvToiwBkgD-?filename=18-022-f152-m%C3%A9dicament%20ENTRESTO-modifi%C3%A9e%20le%204%20juin%202019.pdf
2. McMurray JJ, Packer M, Desai AS, et al. (2014) Angiotensin-neprilysin inhibition versus enalapril in heart failure. *N Engl J Med*;371(11):993-1004.
3. Jhund PS, Fu M, Bayram E, et al. (2015) Efficacy and safety of LCZ696 (sacubitril-valsartan) according to age: insights from PARADIGM-HF. *Eur Heart J*;36(38):2576-84.
4. Ehteshami-Afshar S, Mooney L, Dewan P, et al. (2021) Clinical Characteristics and Outcomes of Patients With Heart Failure With Reduced Ejection Fraction and Chronic Obstructive Pulmonary Disease: Insights From PARADIGM-HF. *J Am Heart Assoc*;10(4):e019238.
5. Seferovic JP, Claggett B, Seidelmann SB, et al. (2017) Effect of sacubitril/valsartan versus enalapril on glycaemic control in patients with heart failure and diabetes: a post-hoc analysis from the PARADIGM-HF trial. *Lancet Diabetes Endocrinol*;5(5):333-340.
